# Estimating proton beam therapy utilization and Investment in Thailand

**DOI:** 10.3389/fpubh.2025.1659275

**Published:** 2025-09-24

**Authors:** Imjai Chitapanarux, Ekkasit Tharavichitkul, Anussara Prayongrat, Patumrat Sripan, Eduardo Zubizarreta, Yavuz Anacak, Pathomphorn Siraprapasiri, Tharatorn Tungkasamit, Chonsanee Changmanee

**Affiliations:** ^1^The Division of Radiation Oncology, Department of Radiology, Faculty of Medicine, Chiang Mai University, Chiang Mai, Thailand; ^2^The Division of Radiation Oncology, Department of Radiology, Faculty of Medicine, King Chulalongkorn Memorial Hospital, Bangkok, Thailand; ^3^Research Institute for Health Sciences, Chiang Mai University, Chiang Mai, Thailand; ^4^Department of Management, International Atomic Energy Agency, Vienna, Austria; ^5^Division of Radiation Oncology, Faculty of Medicine, Ege University, Izmir, Türkiye; ^6^Big Data Institute (Public Organization), Bangkok, Thailand; ^7^National Cancer Institute, Bangkok, Thailand; ^8^Department of Radiotherapy, Maha Vajiralongkorn Thanyaburi Hospital, Prathumthani, Thailand

**Keywords:** proton beam therapy, estimation, utilization, cancer, Thailand

## Abstract

**Background:**

Thailand’s first Proton Beam Therapy (PBT) center became operational in August 2021. Given the high costs and specialized expertise required, evaluating the clinical and economic implications of PBT and planning for its expansion are essential for Thailand’s healthcare system.

**Methods:**

This study projected national PBT demand using data from the Thai PBT center, the Thai Association of Radiation Oncology (THASTRO), national cancer registry reports (Volumes VIII–X), and GLOBOCAN (2022–2040). The number of cancer patients requiring PBT was estimated based on: (1) Thailand’s Comptroller General’s Department (CGD) reimbursement guidelines (June 2023), and (2) the American Society for Radiation Oncology (ASTRO) model policy. Infrastructure, personnel, and cost data were collected, with resource gaps assessed using RRCC version 24.

**Results:**

Between 2022 and 2023, the first PBT center treated 628 patients—approximately 7% of all radiotherapy cases. Under CGD’s limited reimbursement criteria, an estimated 1,454–2,797 patients per year would be eligible for PBT, corresponding to a need for 3–5 PBT units. However, when reimbursement constraints are excluded, this estimate increases to 6–10 units. The ASTRO model suggests even broader eligibility, with 4,471–5,430 patients requiring 10–20 units. The cost of a basic PBT unit is estimated at USD $30 million (excluding building infrastructure), with a treatment course costing approximately $38,000 (RRCC v.24).

**Conclusion:**

The gap between reimbursement- and need-based demand highlights the tension between clinical benefit and cost. Strategic planning must balance equitable access, financial sustainability, and future growth of PBT in Thailand.

## 1 Introduction

Radiotherapy is a cornerstone of cancer treatment. Advances like proton beam therapy (PBT) offer precise targeting and reduced radiation exposure to healthy tissue. Unlike X-ray radiation, PBT uses charged protons that release most energy at a specific depth (the Bragg peak) (1). This allows highly targeted treatment, crucial for tumors near vital organs or in pediatric patients where minimizing radiation is critical. Cancer remains a leading cause of death in Thailand, increasing demand on healthcare resources. The introduction of Thailand’s first PBT center, Her Royal Highness Princess Maha Chakri Sirindhorn Proton Center (HPSP) at King Chulalongkorn Memorial Hospital (KCMH) in Bangkok, marks a significant advancement, reflecting investment in technology and commitment to improving patient outcomes. PBT’s high precision is expected to improve survival, reduce complications, and enhance quality of life.

PBT holds considerable promise for enhancing outcomes and expanding treatment options. Its development is driven by technological advances and clinical trial evidence, though cost assessments are incomplete. As PBT becomes more available, providers must stay updated on clinical data for accurate patient selection (2).

Implementing PBT faces challenges: high costs for purchasing, installation, and maintenance, plus the need for specialized training. PBT’s recent introduction in Thailand necessitates ongoing studies to justify further investment and expansion. This study estimates PBT utilization in Thailand and estimates core investments. This evaluation will provide insights for Thailand’s healthcare system and consider expanding access to this advanced treatment.

## 2 Methods

### 2.1 Estimation of the number of Cancer patients requiring PBT

#### 2.1.1 Patients requiring proton therapy as indicated from the Thailand comptroller general’s department (CGD) reimbursement guidelines (June 2023)

Multiple data sources were used to estimate PBT utilization for diseases eligible for reimbursement in Thailand. These include pediatric tumors (ICD-10-CM C93.30), primary brain tumors (ICD-10-CM C71) based on diagnoses from 2012 to 2018 in the Cancer in Thailand reports (Volumes VIII-X) (3), and patients with unsuitable photon irradiation plans Tumors that cannot be achieved with organs-at-risk (OARs) constraint by photon due to the possibility of life-threatening complications (CTCAE grade 4–5) by consensus of multidisciplinary team based on factors like tumor location, proximity to critical structures, re-irradiation status, and other clinical risks. This was combined with GLOBOCAN (2019–2030) estimated cancer cases (4) and data from the Thai Association of Radiation Oncology (THASTRO) available at https://www.thastro.org/Statistics-Online.php (5). Details on PBT use for these sites were collected from the HPSP. Optimum PBT utilization rates were calculated by determining and summing the frequency of each indication.

#### 2.1.2 Patients requiring proton therapy as indicated from the ASTRO model policy (group 1)

We have followed the methodology of Gupta et al. to quantitatively assess the number of eligible patients and project the demand for particle therapy facilities in Thailand (6). Specifically, we adopted the model proposed by the American Society for Radiation Oncology (ASTRO) to estimate the number of patients eligible for particle therapy (7). This model is based on criteria for medical necessity, published clinical evidence, and disease types that commonly justify the use of particle therapy.

The indications considered include: childhood cancers; malignant and benign primary CNS tumors; advanced and unresectable head and neck cancers (including nasopharynx, nasal cavity, paranasal sinuses, and other accessory sinuses); primary esophageal cancer; malignant pleural mesothelioma; hepatocellular carcinoma and intrahepatic biliary cancers; and advanced or unresectable pelvic tumors with significant pelvic and/or para-aortic nodal involvement.

For pediatric patients, cancer incidence and future trends in Thailand were obtained from the Global Childhood Cancer microsimulation model (8). The proportion of pediatric patients receiving radiotherapy for each diagnosis was derived from Jairam et al. (9). We have assumed that all pediatric patients indicated for radiotherapy are also eligible for proton therapy (10).

For adults (ages 15–85), cancer incidence and future projections from 2020 to 2040 were taken from GLOBOCAN (4). Based on four studies, it is estimated that approximately 13 to 16% of all patients receiving radiotherapy may be eligible for proton therapy (11–14). These estimates were used to calculate the number of eligible adult patients. Additionally, site-specific radiotherapy utilization data were obtained from Delaney et al. (15).

### 2.2 The required proton therapy capacity for Thailand

Data on personnel, machines, staff salaries, and patient numbers were collected from this proton center. These data were entered into the Radiotherapy Resources and Cost Calculator (RRCC), developed in 2014 for the Global Task Force on Radiotherapy for Cancer Control (16). Version 24 (2024) includes proton therapy calculations.

## 3 Results

Based on RRCC guidelines, a proton therapy unit’s annual treatment capacity was estimated using several assumptions: 30 min per fraction (or up to 1.5 h for anesthetized patients), 8 treatment hours per day, 5 days per week, and 50 weeks per year. This schedule yields approximately 4,000 treatment fractions annually. By dividing this number by an average of 15 fractions per patient, the unit has an estimated annual capacity of 267 patient treatment courses. This capacity can be increased with extended operating hours. For example, the PBT unit at KCMH operates on an extended schedule from 6:00 AM to 8:00 PM with two shifts of radiation therapists and a lunch break. This provides 13 treatment hours per day. Under these extended hours, the unit delivers approximately 6,500 treatment fractions annually in maximal estimation, which translates to about 434 patient treatment courses per unit per year. These estimates are consistent with the unit’s actual utilization: 286 patient courses in 2022 and 342 in 2023.

### 3.1 Required proton therapy capacity in Thailand based on CGD reimbursement guidelines (June 2023)

Thailand’s population was 66,052,615 in 2023. From 2022 to 2023, KCMH delivered 8,515 radiotherapy courses, 628 of which used protons. In KCMH, a PBT unit (Varian Pro Beam System) was installed. Proton therapy treatments included 356 courses of proton stereotactic body radiotherapy (SBRT) and 272 courses of intensity-modulated proton therapy (IMPT).

Regarding to the CGD reimbursement guidelines, two categories are eligible for PBT reimbursement: (1) all pediatric cancers (patients under 15 years), and (2) adult cancers for which photon radiotherapy is clinically unsuitable due to unacceptable normal tissue dose. To estimate the national demand for reimbursable PBT, data were analyzed from KCMH, a major academic center providing PBT services. Between August 2021 and October 2024, cancers commonly treated with PBT at KCMH included: Head and neck, bone, and esophageal cancers with unsuitable photon plans or re-irradiation (approx. 17% of cases), Liver cancers (25%), Brain tumors (primarily for re-irradiation, 25%), Pediatric cancers (76% in curative settings), Eye cancers (100% PBT utilization).

To estimate the national number of eligible PBT patients, we used 2022–2023 cancer treatment data from THASTRO, which reported 12,992 patients in 2022 and 14,563 in 2023 across the above cancer types. The average annual patient numbers were multiplied by the observed PBT utilization rates from KCMH to estimate the potential demand.

Regarding reimbursement, 52% of PBT patients treated at KCMH received financial coverage under the Thai government’s reimbursement protocol. However, this figure must be interpreted with caution. While PBT treatment at KCMH began in August 2021, the formal reimbursement framework was only implemented in March 2023. Thus, the 52% rate reflects both eligible patients during the reimbursement period and self-paying patients treated prior to the policy’s introduction. There have been no documented denials of reimbursement when patients met the clinical eligibility criteria. Therefore, this 52% reflects a policy implementation lag rather than limitations of the reimbursement policy itself. This rate was used in the final projection to maintain consistency with available data, resulting in an estimated 1,454 reimbursable PBT patients per year. A breakdown is presented in Table 1.

**Table 1:** Estimated number of proton therapy requirements based on THASTRO data and referenced from KCMH Data.

Disease site	THASTRO 2022	THASTRO 2023	THASTRO Average 22–23	PBT Usage at KCMH	Reimbursement by government *	Estimated number of eligible patients (without reimbursement constraints)	Estimated number of eligible patients (with reimbursement constraints)
Eye	40	99	70	1	0.52	70	36
Pediatric	308	304	306	0.76	0.52	233	121
Liver	1,350	1,481	1,416	0.25	0.52	354	184
Brain	1,236	1,330	1,283	0.25	0.52	321	167
Head and Neck	7,956	8,966	8,461	0.17	0.52	1,438	748
Bone	791	929	860	0.17	0.52	146	76
esophagus	1,311	1,454	1,383	0.17	0.52	235	122
Total	12,992	14,563	13,778	NA	0.52	2,797	1,454

KCMH, King Chulalongkorn Memorial Hospital; THASTRO, Thai Association of Radiation Oncology. *According to the guideline of reimbursement from the CGD, we specified two categories eligible for proton therapy reimbursement: (1) all pediatric cancers (patients under 15 years of age), and (2) other cancers where photon therapy planning is not feasible due to unacceptable overdose to normal tissue. With these indications, 0.52 referred the percentage of patients treated by proton who can be reimbursed from the government at the KCMH.

In the first scenario, based on CGD reimbursement criteria, an estimated 1,454 cancer patients per year would be eligible for PBT. However, if reimbursement limitations are removed and clinical suitability alone is considered, the number increases to 2,797 patients annually (Table 1). Given that one PBT unit can accommodate 267–434 treatment courses per year, this translates to a national requirement of approximately 3–5 PBT units under current reimbursement policy, and 6–10 units if reimbursement constraints are lifted.

### 3.2 Required proton therapy capacity in Thailand based on ASTRO model policy

**Figure 1:**
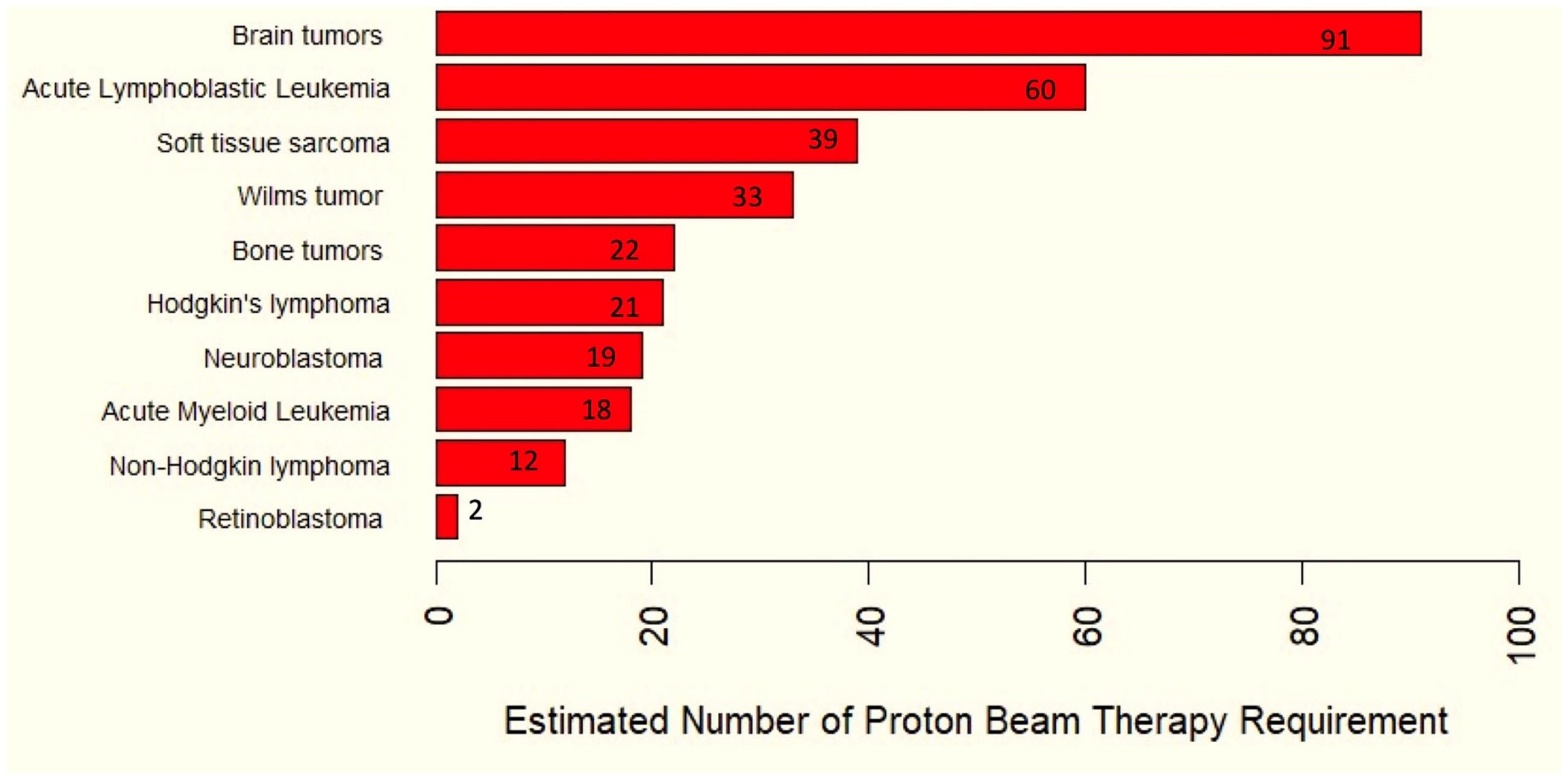
Estimated number of proton therapy requirements for childhood cancers based on as indicated from the ASTRO model policy.

Using the ASTRO model policy (7), it is estimated that 4,471–5,430 cancer patients in Thailand (13–16% of cases) would benefit from PBT (as shown in Table 2 and Figure 1). Based on a unit capacity of 267–434 treatment courses per year, this translates to a national requirement of approximately 10 to 20 PBT units.

**Table 2:** Estimated number of proton therapy requirement for adult cancers based on as indicated from the ASTRO model policy estimated from 2022 to 2040.

Subtypes	RT usage in each diagnosis in % ^a,b^ (15, 19)	Incidence per year 2022 (4)	RT usage in numbers (per year) 2022	Patients eligible for particle therapy in 2022 (13–16% RT)^c^	Incidence per year 2030	RT usage in numbers (per year) 2030	Patients eligible for particle therapy in 2030 (13–16% RT) ^c^	Incidence per year 2040	RT usage in numbers (per year) 2040	Patients eligible for particle therapy in 2040 (13–16% RT) ^c^
Brain, central nervous system	0.92	2,289	2,106	274–337	2,631	2,421	315–387	2,773	2,551	332–408
Head and neck lip, salivary gland, oropharynx, hypopharynx	0.78	6,391	4,985	648–798	7,964	6,212	808–994	8,972	6,998	910–1,120
Nasopharynx	1	2,292	2,292	298–367	2,550	2,550	332–408	2,629	2,629	342–421
Esophagus	0.8	3,147	2,518	327–403	3,886	3,109	404–497	4,320	3,456	449–553
Mesothelioma	1	31	31	4–5	34	34	4–5	38	38	5–6
Liver and intrahepatic bile duct	0.13	26,295	3,418	444–547	32,476	4,222	549–676	36,009	4,681	609–749
Pelvic cancers										
Cervix	0.35	8,354	2,924	380–468	9,368	3,279	426–525	9,679	3,388	440–542
Corpus	0.35	4,191	1,467	191–235	4,729	1,655	215–265	4,830	1,691	220–270
Vulva	0.35	229	80	10–13	287	100	13–16	322	113	15–18
Vagina	0.35	154	54	7–9	186	65	8–10	201	70	9–11
Prostate	0.6	7,042	4,225	549–676	10,140	6,084	791–973	12,244	7,346	955–175
Bladder	0.58	4,105	2,381	310–381	5,572	3,232	420–517	6,602	3,829	498–613
Rectum	0.61	8,529	5,203	676–832	10,755	6,561	853–1,050	12,062	7,358	957–1,177
Anus	0.9	309	278	36–44	396	356	46–57	453	408	53–65
Total		73,358	31,962	4,155–5,114	90,974	39,879	5,184–6,381	101,134	44,556	5,792–7,129

^a^RT usage in each diagnosis from study of Delaney et al. (15). ^b^RT usage in anus from study of Murai et al. (19). ^c^Approximated 13–16% potentially eligible for PT from Gupta et al. (6).

The cost of one simple PBT unit is estimated at 30 million USD (approx. 1,020,000,000 THB, excluding building), with the RRCC v.24 calculating the cost per course at 38,000 USD (1,291,720 THB). (Exchange rate from 1USD to THB on 24th January 2025 = 33.94THB).

## 4 Discussion

Radiotherapy is critical for cancer treatment, and PBT significantly improves tumor targeting and healthy tissue preservation. PBT’s charged protons release energy at the Bragg peak, making it especially beneficial for tumors near vital organs or in pediatric patients. Its adoption represents a significant step in cancer treatment, promising enhanced survival and reduced complications.

Cancer remains a leading cause of mortality in Thailand, pressuring the healthcare system. The establishment of HPSP, Thailand’s first proton therapy center, is a major milestone, representing an investment in advanced technology and a commitment to improving patient outcomes. PBT’s high precision is expected to improve survival rates, reduce side effects, and enhance quality of life.

Despite benefits, PBT’s widespread adoption faces challenges, including high costs for equipment and maintenance, and the need for specialized training. While clinical evidence for PBT grows, cost-effectiveness analyses are incomplete, hindering large-scale investment justification. As a relatively new option in Thailand, ongoing research and outcome assessments are crucial to ensure long-term investment benefits.

This study estimates future PBT utilization in Thailand, focusing on cancers eligible for CGD reimbursement guidelines. Data from THASTRO and KCMH projected the number of patients who could benefit. Projections indicate a substantial increase in PBT demand. Between 2022 and 2023, KCMH treated 628 patients, reflecting increasing utilization.

To estimate required PBT capacity, the study used KCMH data and the RRCC. This tool estimated an annual treatment capacity of 4,000–6,500 fractions per unit from 8–13 working hours. The workload of treatment in KCMH is comparable to the workload of proton therapy in Japan reported by Mizumoto et al. and in Europe by Makbule et al. (17, 18) with an annual capacity of 267–434 treatment courses per unit. This assumes overtime shifts could accommodate additional patient volume. Each PBT unit costs about 30 million USD, with a calculated cost per course of 38,000 USD.

The estimated national need for PBT units in Thailand varies significantly depending on reimbursement policies. Under the CGD guidelines (June 2023), which restrict coverage to select pediatric and adult cases with strong evidence, about 1,454 patients annually are eligible, requiring 3–5 PBT units. Without reimbursement limits, eligibility nearly doubles to 2,797 patients, increasing the projected need to 6–10 units. Retaining both estimates offers a balanced view, providing policymakers and stakeholders with valuable insights for strategic planning and resource allocation.

In contrast, a broader clinical need–based model, guided by international evidence such as the American Society for Radiation Oncology (ASTRO) model, includes a wider range of indications with potential benefit from proton therapy and accounts for rising cancer incidence (7). Under this approach, the estimated national requirement increases substantially to 10–20 units. The gap between these two estimates highlights the difference between policy-driven (reimbursement-based) and clinically driven (need-based) demand.

The CGD model reflects a more conservative, cost-conscious stance aligned with current healthcare financing structures, while the need-based model anticipates long-term demand growth and aims to optimize clinical outcomes. Future planning for proton therapy infrastructure will need to balance these perspectives to ensure equitable access, financial sustainability, and clinical benefit across Thailand’s cancer care system.

This study provides an initial estimate of the number of patients in Thailand potentially eligible for reimbursed proton beam therapy (PBT), based on clinical need and current reimbursement policies. However, several limitations must be considered when interpreting these findings. First, the 52% reimbursement rate used in our projections may underrepresent the actual alignment between clinical eligibility and financial coverage. Proton therapy services at KCMH began in August 2021, while the national reimbursement policy only became effective in March 2023. Therefore, many patients treated before this policy were self-funded despite meeting clinical criteria. As such, the 52% figure does not imply that 48% of patients were deemed ineligible by the reimbursement system; rather, it reflects a transition period during which policy coverage had not yet been fully implemented. Second, to date, there is no evidence of reimbursement rejection for clinically eligible patients under the current CGD criteria. In practice, when a clinician deems a photon plan unsuitable and submits appropriate documentation, reimbursement has been granted. Thus, the actual rate of future reimbursement may be significantly higher than what is currently observed. Despite these limitations, the 52% rate was conservatively applied to estimate the number of reimbursable cases, providing a cautious but grounded projection. As the policy continues to mature and retrospective funding is clarified, the proportion of eligible and reimbursed patients is expected to increase. Lastly, this study relies on institutional data from a single academic center. National databases currently lack fields such as “re-irradiation” or “unsuitable photon plan,” which are critical for determining PBT eligibility. Future efforts to standardize such clinical indicators at the national level will help refine these estimates and support more accurate planning for proton beam therapy services.

Despite these limitations, the study offers valuable insights into PBT use in Thailand. Furthermore, RRCC version 24 facilitated calculations for required competency, capital cost, and per-unit cost, informing projections for Thailand’s PBT unit needs. Future models may vary based on factors like increased hypofractionation or different proton indications.

These projections highlight the growing need for PBT in Thailand and the substantial investments required to expand access. The findings suggest that with proper infrastructure and continued research, PBT could be integral to improving cancer care. However, careful consideration of costs, patient outcomes, and healthcare system sustainability is necessary to ensure PBT remains viable and effective.

## 5 Conclusion

Our findings suggest that expanding PBT infrastructure in Thailand will require a strategic balance between financial sustainability, equitable access, and clinical effectiveness. While current CGD reimbursement guidelines support a limited set of indications, broader clinical evidence points to a much higher potential need. Aligning policy with emerging clinical data is essential to meet growing demand. Despite some data limitations, this study offers a strong foundation for future planning. With targeted investment and evidence-based reimbursement strategies, PBT can become a key component of cancer care in Thailand.

## Data Availability

Publicly available datasets were analyzed in this study. For THASTRO, this data can be found at: https://www.thastro.org/Statistics-Online.php. For the GLOBOCAN data, these data can be found at: https://gco.iarc.fr/today/en and https://gco.iarc.fr/tomorrow/en, respectively.
